# Tunable Work Function and Surface Energy in Titanium Nitride (TiN) Thin Films through Quantum Well States

**DOI:** 10.1021/acsmaterialsau.4c00176

**Published:** 2025-01-20

**Authors:** Angus Huang, Yee-Heng Teh, Chin-Hsuan Chen, Sheng-Hsiung Hung, Jer-Fu Wang, Chih-Piao Chuu, Horng-Tay Jeng

**Affiliations:** † Department of Physics, National Tsing Hua University, Hsinchu 30013, Taiwan; ‡ Physics Division, National Center for Theoretical Sciences, Taipei 10617, Taiwan; § Center for Theory and Computation, National Tsing Hua University, Hsinchu 30013, Taiwan; ∥ Corporate Research,Taiwan Semiconductor Manufacturing Company Limited, Hsinchu 30091, Taiwan; ⊥ College of Semiconductor Research, National Tsing Hua University, Hsinchu 30013, Taiwan; # Institute of Physics, Academia Sinica, Taipei 11529, Taiwan; ∇ Research Center for Semiconductor Materials and Advanced Optics, Chung Yuan Christian University, Taoyuan 32031, Taiwan

**Keywords:** semiconductor, TiN, workfunction, first-principles, quantum well state

## Abstract

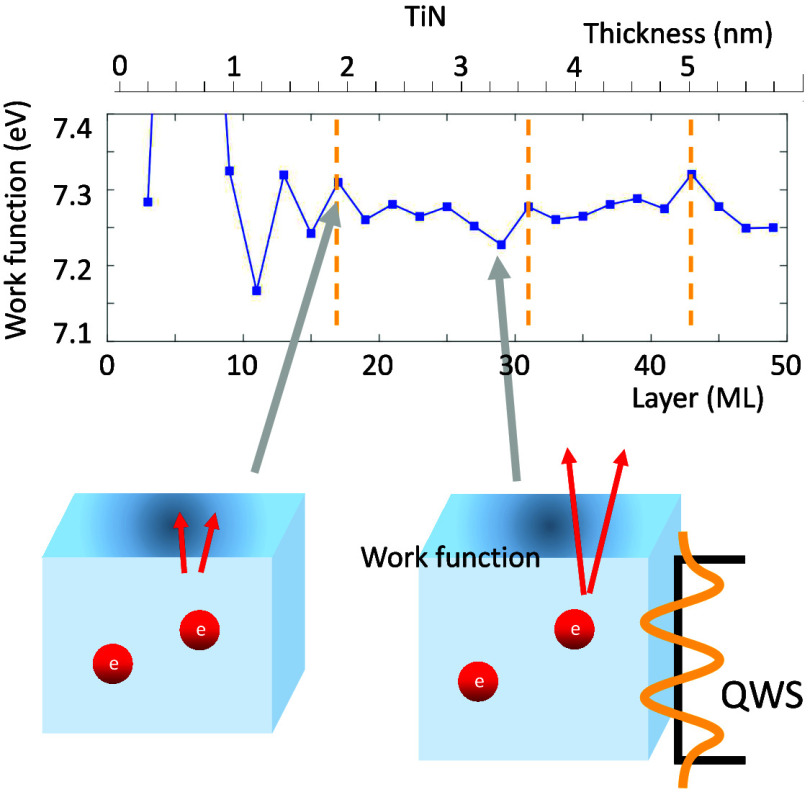

High work function metals are crucial in various semiconductor applications. Titanium nitride (TiN) is particularly noteworthy as a high work function material in metal gate structures, which significantly enhances the transistor performance and reliability in advanced semiconductor devices. In this study, we employ first-principles calculations to demonstrate that the TiN work function oscillates with thickness due to the quantum well state effect. Furthermore, we investigate the termination and surface dependence of the work function across different crystallographic orientations. We show that the work function can be enhanced to up to 8.04 eV for TiN(111) with N-termination at five monolayers (5 MLs). Our findings provide valuable insights for fine-tuning the high work function of TiN.

## Introduction

Transition metal carbides and nitrides (TMCNs), specifically MX compounds (where M is a transition metal and X = N, C), particularly those with a rocksalt (NaCl) structure, exhibit various unique properties. These include energy storage,^1^ superconductivity,^2−4^ topological superconductivity,^5^ and remarkable thermal stability. The rocksalt structure of TMCNs impedes ion migration and leads to the exceptional thermal stability, extremely high melting point,^6^ and hardness,^7−9^ making them highly desirable for industrial applications such as wear-resistant coatings.^6,10^ Notably, high work function^11,12^ and thermal stability renders titanium nitride (TiN) an ideal material for metal gate components in metal–insulator–metal (MIM) integrated capacitors and complementary metal-oxide-semiconductor (CMOS) technologies. Although previous first-principle studies^6,12−15^ have elucidated formation energies and work functions of TiN thin films, these properties still require clarification for TiN ultrathin films.

Quantum well state (QWS) is a two-dimensional (2D) electronic state confined within a thin film by boundaries such as vacuum, cap layer, and substrate. An increasing or decreasing in film thickness can cause the QWSs to intersect the Fermi level (E_*F*_), leading to significant changes in density of states (DOSs) near E_*F*_. The periodic changes in DOS along with varying film thickness^16^ are crucial for various properties including thermal stability,^17^ effective mass,^18,19^ work functions,^16,20−22^ surface energy,^16,21−23^ interlayer exchange coupling,^24^ and the superconducting transition temperature,^25,26^ magnetic anisotropy,^27^ as evidenced by prior experiments and theoretical studies. Owing to the continuous reduction in sizes of devices, it is necessary to seriously investigate the effects of QWS in TiN thin films.

It is noteworthy that the impact of quantum well states on TiN thin films for advanced semiconductor devices has not been reported to date. In this work, we investigate the QWS effect in TiN thin films with N- and Ti-terminations of (001), (011), and (111) surfaces for slab thickness from a few atomic layers up to 6 nm through *ab initio* simulations. Our results reveal that the work function of TiN(111) thin film with N-termination (label as “TiN(111)N”) is approximately 7.30 eV with the maximum work function of 8.04 eV at 5 ML, which is notably higher than previously reported values for TiN systems ranging from 4.1 to 5.3 eV depending on growth conditions.^10−13^ We demonstrate that the work function, surface energy, and DOS at E_*F*_ of TiN slabs oscillate periodically with a thickness larger than 2 nm. For ultrathin films with thicknesses ≲2 nm, the oscillation is even more drastic. Analysis on band structures of various TiN slabs reveals that the QWSs derived from the quantum confinement effect serve as the driving force of the thickness-dependent oscillations of these properties. The origins of the high work function and the stability of different terminations are also discussed.

## Method

First-principles electronic structure simulations are performed using the Vienna Ab initio Simulation Package (VASP)^28−30^ based density functional theory (DFT). The projector-augmented-wave (PAW)^31,32^ pseudopotentials with generalized gradient approximation (GGA) exchange-correlation functionals in the Perdew–Burke–Ernzerhof (PBE)^33^ revised form for solids (PBEsol)^34^ are adopted in this work. For (111), (011), and (001) slabs, we used vacuum layers of thickness 15, 18, and 12.5 Å, respectively, in the calculations. Additionally, we have examined the convergence of the vacuum layer thickness and found that when the vacuum layer exceeds 12 Å, the changes in the work function and surface energy become negligible. The lattice structures of TiN slabs are optimized using PBEsol functional until the atomic force is less than 0.02*eV*/*Å*. The **k**-point grid of 24 × 24 × 1 and energy cutoff of 400 eV are used in self-consistent calculations. We first calculate work functions using VASP as well as QuantumATK^35,36^ and confirm they yield same results. Then we employ QuantumATK to perform Green’s function surface calculation^37^ using the norm-conserving pseudopotential linear combination of atomic orbitals (LCAO) basis set approach.^38^ The results are shown in Table S1, where we can see that QuantumATK and VASP give similar work functions for all four surface orientations.

Force constant and phonon band structure calculations are performed based on the density functional perturbation theory (DFPT).^39^ Quantum Espresso^40^ packages are employed with ultrasoft pseudopotentials^41^ using local-density approximation (LDA)^42,43^ functional for phonon calculations. The 8 × 8 × 8 **k**-grid and 4 × 4 × 4 **q**-grid are used for TiN unit cell in phonon simulations. The energy cutoff is 40 Ry (400 Ry) for wave function (charge density) calculations. The 0.02 Ry broadening is set in the Fermi–Dirac distribution for phonon mode calculations.

To obtain the surface phonon spectrum, similar to previous studies,^44,45^ we start from phononic Green function formula^46^
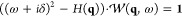
1with phonon momentum **q** and energy ω. Here *H*(**q**)  is the phonon Hamiltonian (Green function) and the δ is a small energy broadening. The phonon spectrum  is simulated using
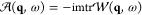
2The surface phonon  and bulk phonon  spectra are calculated based on the force constant and Sancho Rubio method^47^ derived from DFPT.

## Results and Discussion

**Figure 1:**
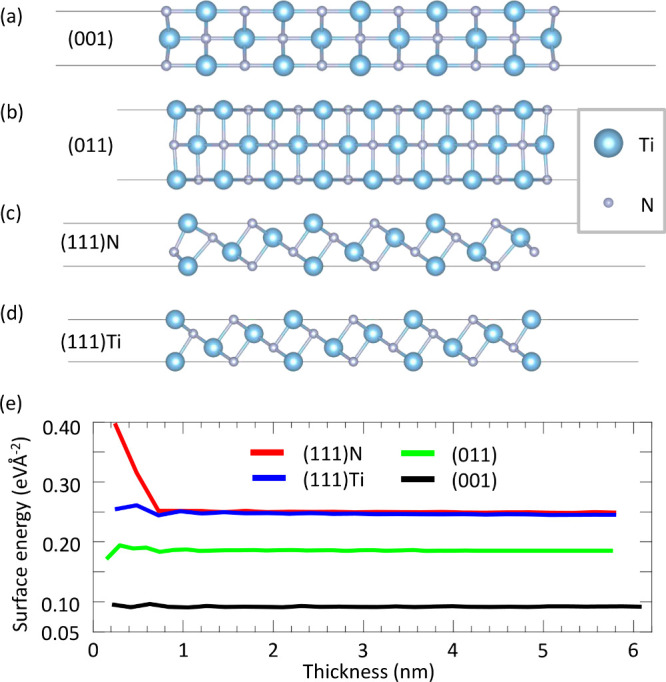
(a–d) Side view of DFT-relaxed TiN slabs with different surface orientations. (a) TiN(001). (b) TiN(011). (c) TiN(111) N-terminated (TiN(111)N). (d) TiN(111) Ti-terminated (TiN(111)Ti). (e) Surface energies of TiN as functions of slab thickness for different surface orientations.

Bulk TiN possesses a cubic rocksalt structure with a space group of Fm3̅m. The calculated lattice constant of 4.19 Å using the PBEsol functional is in good agreement with the experimental value of 4.24 Å.^48^ Among various surface orientations, we considered three main directions (001), (011), and (111) of TiN thin films, as illustrated in Figure 1(a)-(d). For the TiN(001) and TiN(011) surfaces, each has a single type of surface only. While for TiN(111), two different types of cutting surfaces, N-terminated (TiN(111)N) and Ti-terminated (TiN(111)Ti) are shown in Figure 1(c) and (d), respectively. To facilitate the estimation of physical properties, we adopt TiN(111) slabs with odd-number atomic layer thicknesses to ensure that the two surfaces of the (111) slab are the same.

To determine the thickness-dependent surface stability of various surface orientations and faces, we calculated the surface energy *E*_surf_ of each TiN thin film via
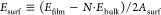
3Here *E*_film_ (*E*_bulk_) is the total energy of the whole thin film system (TiN bulk unit cell), *N* represents the ratio of the number of atoms in the thin film to that in the bulk material, and *A*_surf_ is the surface area of the thin film. Calculated surface energies of geometrically optimized TiN thin films are listed in Figure 1(e). For all considered TiN surfaces, the surface energy converges quickly when the thickness is greater than 2 nm with a very small fluctuation less than 0.01 eV/Å^2^. For (001) and (011) surfaces, the surface energies eventually converge to 0.09 and 0.18 eV/*Å*^2^, respectively, being consistent with 0.08 and 0.18 eV/*Å*^2^ from previous DFT calculations.^15^ For (111)Ti and (111)N surfaces, the surface energies are very close to each other, with the former slightly lower than the latter by ∼0.05 eV/*Å*^2^. The converged surface energies with respect to the film thickness for (111)Ti and (111)N are 0.245 and 0.250 eV/*Å*^2^, respectively, which are also in good agreement with 0.31 eV/*Å*^2^ for (111)Ti from previous DFT results.^15^ As for the ultrathin film limit, the surface energy shows significant deviations in contrast to the well-converged results for the thick film limit in all the cases studied, particularly the (111) surface. Such enhanced fluctuation in ultrathin films originates from the quantum well state due to the quantum confinement effect and provides a valuable opportunity to manipulate high work function metal electrodes, as will be discussed later.

**Figure 2:**
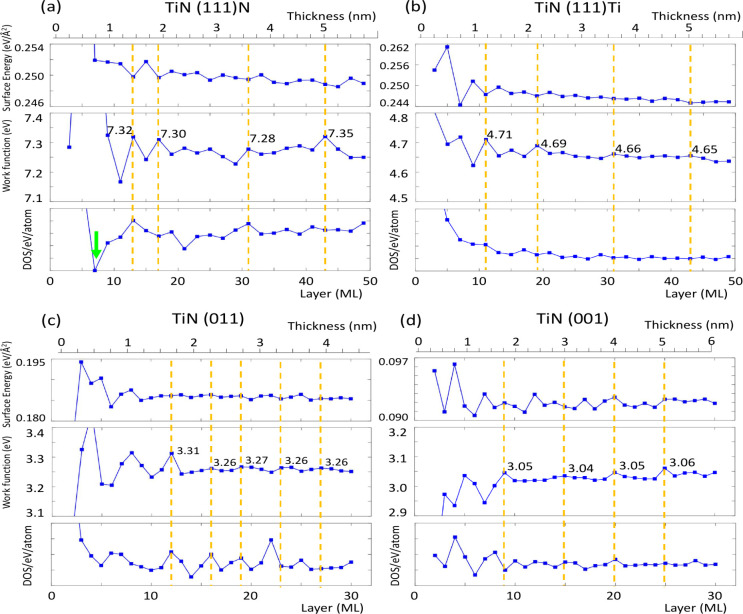
Thickness dependence of surface energy, work function, and DOS per atom at the Fermi level of TiN thin films with different surface orientations and faces from DFT calculations. (a) For TiN(111)N. (b) For TiN(111)Ti. (c) For TiN(011). (d) TiN(001). The green arrow in (a) indicates a semiconducting state for 7 ML (see Figure S1). Each ML contains one Ti and one N atom in the unit cell. Orange dashed lines highlight the relatively high work functions of TiN slabs. Due to the quantum well state effect, strong fluctuation emerges in ultrathin TiN films with thickness less than 2 nm.

To illustrate the periodic behavior of surface energy (*E*_*surf*_) and work function (W) and reveal the intimate interplay with QWS of TiN thin films, we present layer-by-layer analyses of *E*_*surf*_, W, and DOS at the Fermi level (N(E_*F*_)) in Figure 2 with the QWS band structures shown in Figures S1–S4. For the (111)N surface (Figure 2(a)), the calculated work function around 7.3 eV is significantly higher than that reported in previous experimental results. In contrast, the (111)Ti surface displays a work function close to 4.7 eV (Figure 2(b)) in line with previous DFT and experimental findings. Both TiN surfaces show clear periodic variations in electronic structures similar to the Pb(111) thin films.^16,21^ For (111)N thin films with thickness thicker than 2 nm, the period of relatively high work functions is about 1.5 nm (12–14 MLs) as highlighted by the orange dashed lines. For thicker (111)Ti films, the surface energy and work function (Figure 2(b)) also exhibit periodicity comparable to those of the (111)N thin films. Similar to previous QWSs studies on Pb thin films,^16,21^ the oscillations in surface energy and work function are also out-of-phase for both the TiN(111)Ti and TiN(111)N thin films as shown in Figure 2(a) and (b), respectively. While for ultrathin films less than 2 nm, the period of variation is irregular. Moreover, the periodic character can also be found in (001) and (011) orientations as presented in Figure 2(c) and (d) with shorter periods of ∼1 nm (5 ML) and ∼0.5 nm (2.5 ML), respectively. Such a general periodic behavior is originated from the QWS that passes through the Fermi level one by one along with changing the film thickness due to the quantum confinement effect.^16^ Band structures of TiN thin films with various directions and thicknesses presented in Figure S1 elucidate the close relation among the QWSs and electronic structures investigated in the work.

**Figure 3:**
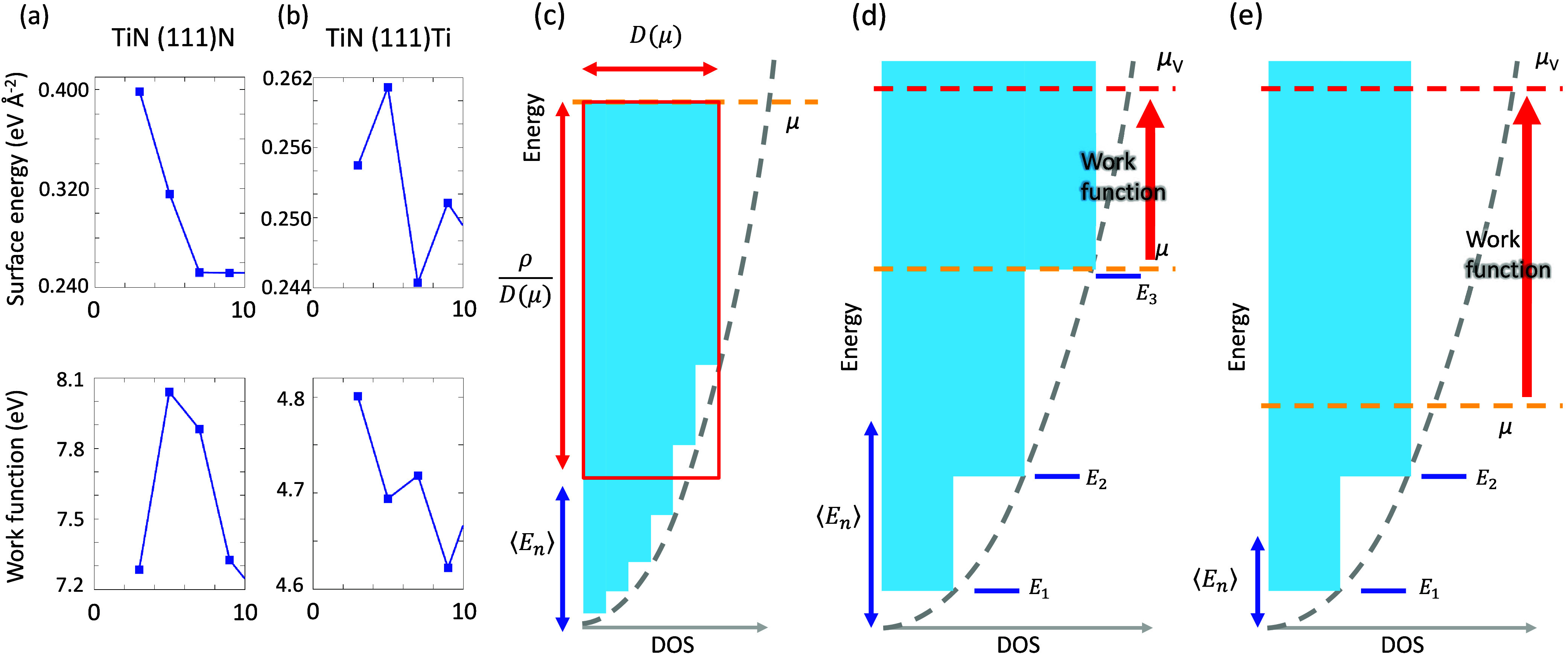
(a,b) Zoom-in plots of Figure 2(a),(b) for TiN(111)N and TiN(111)Ti ultrathin films with thickness less than 1 nm, respectively. (c–e) Schematic illustration of how thickness-dependent QWSs affect the Fermi level and work function. Light blue regions indicate the DOS contributed by QWSs. The chemical energy μ and the vacuum level μ_V_ are indicated by orange and red dash lines, respectively. Gray curves indicate the quadratic-like DOS of two-dimensional free electron systems. ⟨*E*_*n*_⟩ denotes the average energy of occupied QWSs. The area of the red box approximates the electron density ρ.

It can also be seen in Figure 2 that the electronic properties change drastically for TiN thin films with thicknesses less than 2 nm, especially less than 1 nm. Figure 3(a) and (b) provides magnified results for TiN(111) ultrathin films with thicknesses below 1 nm. For the (111)N surface, the work function rapidly increases, reaching 8.04 eV at 5 MLs, then drops back to 7.28 eV at 3 ML. Additionally, a phase transition into a semiconductor occurs at 7 ML before reverting to the metallic state at 5 ML (Figure 2(a) and Figure S1). For the (111)Ti surface, the work function rapidly increases and reaches the peak value of 4.08 eV at 3 ML. We note that the abrupt changes in electronic properties of Pb ultrathin films have also been observed in experimental measurements as well as in theoretical predictions.^21,23^ However, a reasonable explanation is still lacking. Here, we propose a systematic picture for this conspicuous behavior in TiN ultrathin films based on QWSs and quantum confinement effect as discussed below, which serves as the foundation of TiN as well as Pb ultrathin films.

The electron density (ρ) in a two-dimensional free electron system with a quadratic-like DOS (Figure 3(c-e)) can be approximated by the product of DOS at E_*F*_ (D(μ)) and the chemical potential (μ), i.e., ρ ≃ *D*(μ)·μ + const. as schematically plotted in Figure 3(c). Therefore, the chemical potential (Fermi level) can be expressed as^16^
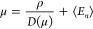
4where ⟨*E*_*n*_⟩ denotes the average energy of occupied QWSs. Work function (W) is the energy needed to move an electron from the Fermi level to vacuum, i.e.,
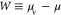
5where μ and μ_v_ are the Fermi level and vacuum level, respectively. For two-dimensional QWS systems, the DOS appears as a series of stacked step functions (Figure 3(c)-(e)). As long as the film thickness increases, the quantum confinement is relaxed, and hence the QWS energy decreases. Once an extra QWS crosses the Fermi level, *D*(μ) suddenly increases, and the first term in eq 4 abruptly decreases, resulting in rapid decrease in μ. Although ρ in the numerator of the first term and ⟨*E*_*n*_⟩ in the second term in eq 4 are also varying along with changing the film thickness, *D*(μ) in the denominator has a stronger effect to decrease μ. Consequently, the work function (eq 5), i.e., the chemical potential relative to the vacuum level, can be enhanced significantly^16^ as depicted in Figure 3(c)-(e). Additionally, since the energy difference between the levels of QWSs is inversely proportional to the thickness of the thin film, such variations have a more pronounced effect on the work function of ultrathin films. The above discussion demonstrates that QWS systems, such as Pb and TiN thin films, exhibit extreme characteristics, especially when they are ultrathin.

**Figure 4:**
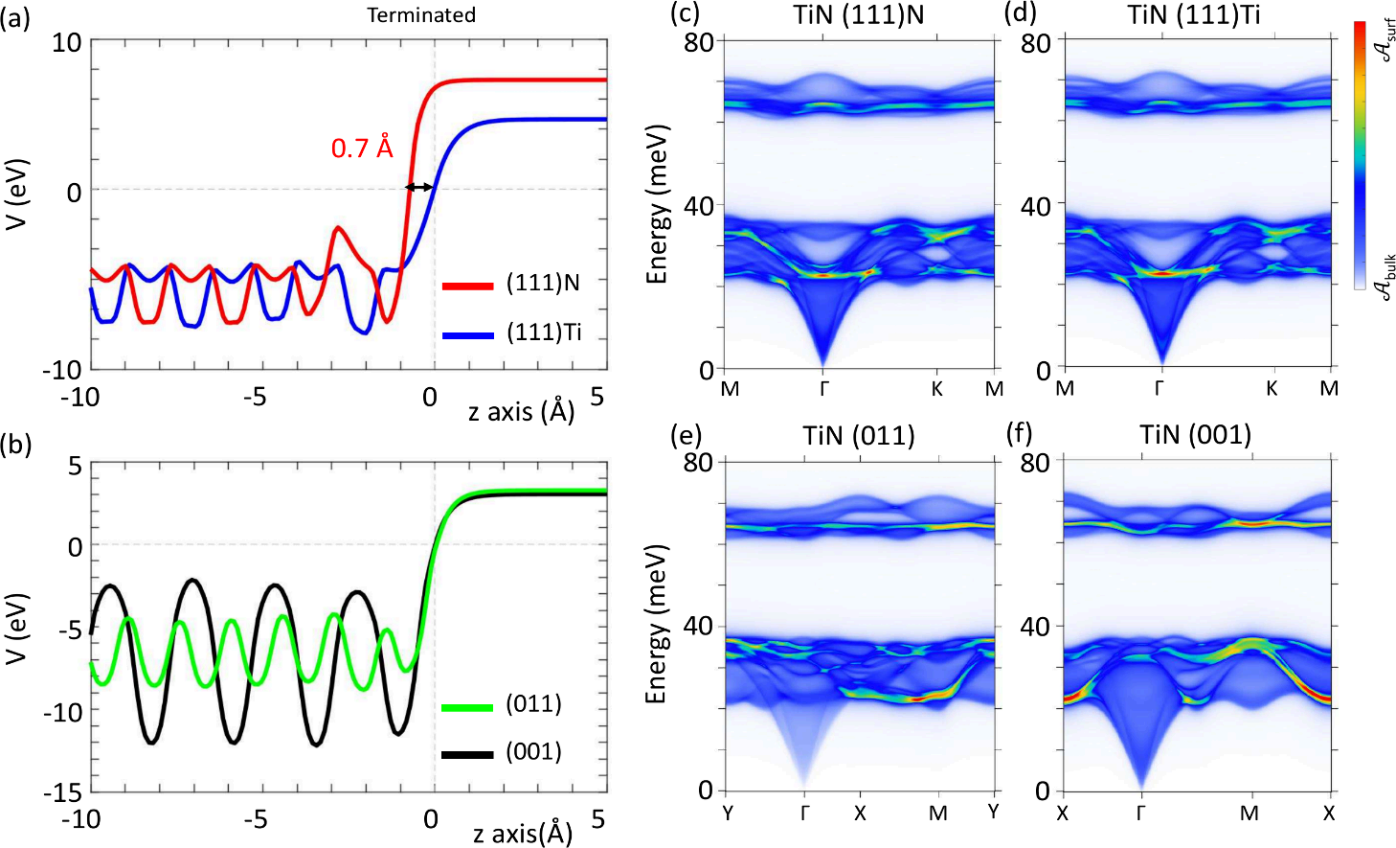
(a,b) Potential profiles along the surface-normal direction (*z*) for various surfaces of TiN thin films with thickness of 3 nm. The Fermi level is at the zero energy. In (a), the inner layers of (111)N and (111)Ti thin films are aligned together for a clear comparison. In (b), zero potential points are shifted to *z* = 0 for simplicity. (c–f) Decomposed phonon band structures of various TiN thin films. Red (blue) color indicates surface (bulk)-dominant phonon bands.

To clarify why different facets of TiN result in significantly different work functions shown in Figure 2, we depict the potential profiles along the surface-normal (*z*) directions of TiN thin films in Figure 4(a) and (b). By aligning the inner layers of the relaxed lattice structure of (111)N and (111)Ti slabs in Figure 4(a), we reveal a significant surface contraction in the (111) N-terminated surface potential by 0.7 Å in comparison with the (111) Ti-terminated surface potential. Such a dramatic behavior in (111)N surface is induced by the notable surface layer contraction of 0.12 Å in the surface N-layer with respect to the surface Ti layer in (111)Ti surface. In addition to the relatively strong electron negativity of N, the work function of (111)N thin film is thus much higher than that of (111)Ti thin film. These achieve a steep surface potential for (111)N, which in turn induces a substantial work function in comparison with the (111)Ti surface. The potential profiles in (001) and (011) surfaces are smoother as compared with the (111) cases and thus lead to much lower work functions as shown in Figure 4(b).

To study the stability of these four surfaces, we employ the Sancho-Rubio method^47^ to calculate the surface phonon green function of different TiN facets as presented in Figure 4(c)-(f). It can be seen that all four TiN facets: (111)N, (111)Ti, (001), and (011), exhibit stable lattice structures without any negative phonon. Furthermore, we identify surface contributions to the phonon band structure through  (Method section) with surface components highlighted by different colors, so as to be distinguished from bulk contributions  depicted in blue and gray. Overall, the four facets show similar phonon band structures, including band energies and band dispersions. But the surface component distributions are different from each other. For example, the surface component distributes similarly for the (111)N and (111)Ti surfaces, showing a nearly flat optic surface band and a major acoustic surface band with the band minimum at ∼22 meV around Γ. Whereas the major acoustic surface band in (011) and (001) surfaces displays the band minimum at ∼22 meV around M and X, respectively. In any case, the surface phonons exhibit no imaginary frequencies, concluding that all of these facets of TiN are stable and can be realized in experiments.

To demonstrate that both the TiN(111)Ti and TiN(111)N thin films can be synthesized in spite of relatively high surface energies (Figure 1(e)), we further calculated the formation energy *E*_form_ of TiN(111) slabs (14MLs) with respect to the growth condition as presented in Figure S5. Following the standard approach^49^ by treating the excess Ti/N as surface defects, the formation energy is calculated through
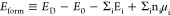
6where *E*_D_ is the total energy of the defect-containing (111) surface (i.e., Ti/N termination at both sides of surfaces), *E*_0_ is the total energy of the (111) surface with Ti/N-termination at opposite surfaces, *E*_*i*_ is the total energy per atom of element *i* in referenced configuration, *n*_*i*_ is the number of atoms changed during the formation of the defect, and μ_*i*_ is the chemical potential of element *i*. In this study, the hcp phase of Ti metal and the gas phase of Nitrogen are chosen to be the referenced configuration of Ti and N, respectively. The chemical potentials μ_Ti_ and μ_N_ are the range of values that satisfy the equation μ_Ti_ + μ_N_ = Δ*E*(TiN), where Δ*E*(TiN) = −3.08 eV is the formation energy per formula unit of TiN slab with respective to the referenced configuration of Ti and N. The calculated results presented in Figure S5 show that both the (111)Ti and (111)N surfaces are energetically favorable in Ti-rich and N-rich environments, respectively, and thus can be synthesized experimentally by suitably controlling the growth conditions. Our findings that TiN thin films exhibit high work function at specific thicknesses or crystallographic orientations are potentially applicable in optimizing the fabrication process of TiN thin films such as in the surroundings of nitrogen to prevent oxidation and enhance the work function at the same time.

**Figure 5:**
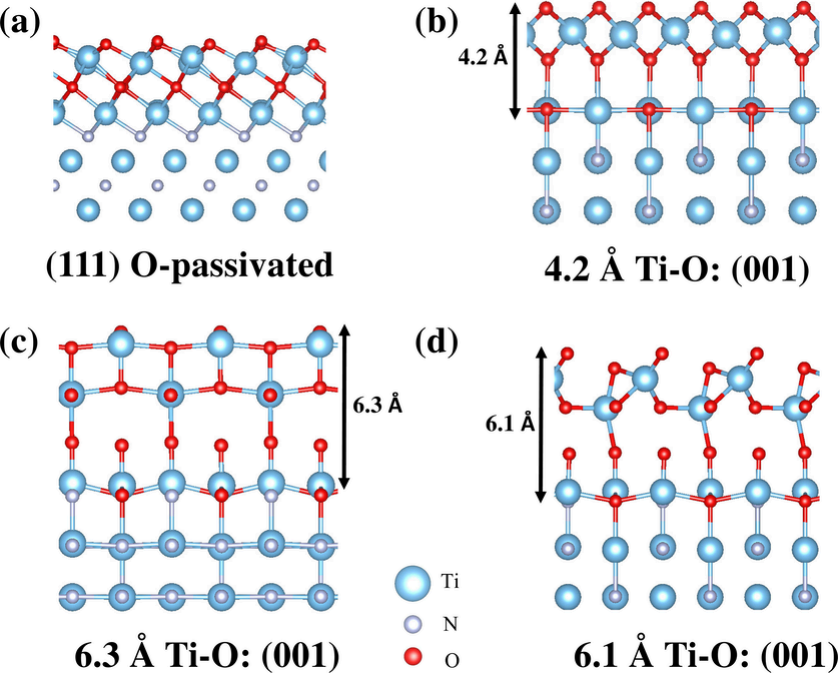
Side view of optimized (a) TiN(111) O-passivated structure. (b–d) TiN(001) structure stabilized with Ti–O capping layer with various thicknesses of 4.2, 6.3, and 6.1 Å, respectively.

It is worth noting that previous DFT studies^12^ have indicated that TiN is prone to oxidation, with experimental measurements often reflecting the influence of oxidation and defects.^12^ To investigate the impact of oxidation on work function, we simulate the TiN(111)N slab using a 2 × 2 supercell with the thickness of ∼2 nm, as shown in Figure 5(a). In this model, we substituted the outermost and second outermost layers of N atoms with O atoms on both sides of the slab and optimized the lattice structure. The defect formation energy was calculated using Equation 6, where the chemical potential of oxygen (μ_*O*_) was taken as the total energy per atom of molecular oxygen gas. The calculated defect formation energy per *n*_*i*_ ranges from −3.05 eV/N_i_ to −1.48 eV/N_i_, depending on the choice of N chemical potential, where N_i_ represents the number of atoms added or removed from the pristine TiN(111)N slab. This oxidized TiN(111)N model exhibits a work function of 5.45 eV, which is close to the experimental range (4–5 eV).^50−52^ To further explore the effects of the O vacancies, we removed one O atom from the top layer of the slab in Figure 5(a). The defect formation energy becomes less negative, ranging from −2.86 eV to −1.78 eV, while the work function decreases to 5.01 eV, agreeing well with experimental data. These highly negative defect formation energies suggest that both the TiN(111)N and TiN(111)Ti structures are susceptible to oxidation with their surfaces effectively passivated by O atoms, leading to the work function observed in experiment.

Next, we simulate the oxidation effects of the TiN(001) slab using a  supercell with an approximate thickness of 2 nm. Following approaches similar to previous studies,^12,53^ we observed that oxidized surfaces rearrange into different configurations depending on the number and initial positions of O atoms. We present three different configurations of the oxidized (001) slab in Figure 5(b)-(d), each stabilized by a single Ti–O capping layer with thicknesses of 4.2, 6.3, and 6.1 Å, respectively. Their formation energies and work functions are summarized in Table 1. Among these configurations, the 4.2 Å Ti–O: (001) structure exhibits the lowest formation energy with the work function of 5.1 eV that closely matches experimental values. Overall, the highly negative defect formation energies suggest that the (111)N, (111)Ti and (001) surfaces are highly susceptible to oxidation. This susceptibility provides a plausible explanation for the low work function values observed in the experimental measurements.

**Table 1:** Range of Defect Formation Energy per *n*_*i*_ and Work Function of Oxidized TiN(001) Slab^a^

	4.2 Å Ti–O: (001)	6.3 Å Ti–O: (001)	6.1 Å Ti–O: (001)
Minimum formation energy per *n*_*i*_ (eV)	–3.18	–2.84	–2.78
Maximum formation energy per *n*_*i*_ (eV)	–1.97	–1.74	–1.69
Work function (eV)	5.10	6.43	8.8

a The minimum and maximum formation energies were calculated using eq 6 with the lower and upper limits of nitrogen’s chemical potential, respectively.

The high work function of TiN(111)N slab found in this work suggests its high potential as an electrode interface with p-type semiconductors to facilitate efficient hole injection mechanisms^54−58^ or the metal gate in MOSFET to control the threshold voltage.^59−62^ TiN thin films synthesized to this end are thus protected from oxidation by the semiconductor substrate that can properly seal the TiN interface and effectively inhibit the diffusion of water and oxygen molecules into TiN thin films. Furthermore, our calculations indicate that the work function of the TiN surface on one side remains largely unaffected by the surface termination on the opposite side, as illustrated in Figure S6 Thus, appropriate oxidation prevention measures, such as metal plating, chemical conversion coatings, and polymer coatings^63−66^ can be applied to the TiN surface exposed to air. This study highlights the importance of further research into the physics of various TiN-semiconductor heterostructures, particularly focusing on the energy barriers for hole and electron injection and the stability of the interface.

## Conclusion

In summary, this work demonstrates that the work function and surface energy of TiN exhibit oscillations along with changing film thickness because of the quantum well states (QWSs) similar to those observed in Pb thin films. Given TiN’s critical role in semiconductor devices, these oscillations may have significant implications for implementations. Furthermore, our results reveal a dramatic change in electronic properties of TiN ultrathin films especially when the film thickness is less than 1 nm. Notably, N-terminated TiN(111) thin films exhibit a high work function around 7 eV and can be as high as 8.04 eV at 5 monolayers (MLs). Our findings pave a new route for manipulating high work functions of TiN thin films by optimizing the manufacturing of semiconductor devices via, for example, layer-by-layer growth under well-controlled synthesis conditions such as processing in the nitrogen-rich chambers.

## Data Availability

The data that supports the findings of this study are available within the article and Supporting Information.

## Supporting Information Available

Band structures of TiN slabs with various thicknesses and terminations; surface formation energy under different conditions; potential and work function profile along the surface-normal direction; work functions of TiN at different surface orientations (PDF)
