# Supplementary material for: Tunable Work Function and Surface Energy in Titanium Nitride (TiN) Thin Films through Quantum Well States
Source: ACS Mater Au. 2025 Jan 20;5(2):430–7. doi: 10.1021/acsmaterialsau.4c00176 (PMC11907290; doi:10.1021/acsmaterialsau.4c00176)
Supplement: Supplementary file 1 — mg4c00176_si_001.pdf [file mg4c00176_si_001.pdf]

Supporting Information

Tunable work function and surface energy in titanium nitride (TiN) thin films through quantum well states

Angus Huang†,[1,2,3] Yee-Heng Teh†,[1] Chin-Hsuan Chen,[1] Sheng-Hsiung Hung,[1]
Jer-Fu Wang,[4] Chih-Piao Chuu,[4] and Horng-Tay Jeng*[1,2,5,6,7]

[1]*Department of Physics, National Tsing Hua University, Hsinchu 30013, Taiwan*
[2]*Physics Division, National Center for Theoretical Sciences, Taipei 10617, Taiwan*
[3]*Center for Theory and Computation, National Tsing Hua University, Hsinchu 30013, Taiwan*
[4]*Taiwan Semiconductor Manufacturing Company Limited, Hsinchu 30013, Taiwan*
[5]*College of Semiconductor Research, National Tsing Hua University, Hsinchu 30013, Taiwan*
[6]*Institute of Physics, Academia Sinica, Taipei 11529, Taiwan*
[7]*Research Center for Semiconductor Materials and Advanced Optics,*
Chung Yuan Christian University, Taoyuan 32031, Taiwan

†These authors contributed equally to this work.
* jeng@phys.nthu.edu.tw

KEYWORDS: semiconductor, TiN, workfunction, first-principles, quantum well state

Content:

Fig. S1. Band structure of TiN (111) N-terminated thin film.
Fig. S2. Band structure of TiN (111) Ti-terminated thin film.
Fig. S3. Band structure of TiN (011) thin film.
Fig. S4. Band structure of TiN (001) thin film.
Fig. S5. Formation energy of surface with N-termination at both sides, (111) N; surface with Ti-termination at both sides, (111) Ti; surface with Ti/N-termination at opposite sides, (111)Ti/(111)N.
Fig. S6. Potential profile along the surface-normal direction (left axis) and work functions (right axis) of a (111) slab with N-passivated and O-passivated surfaces at opposite ends.
Table. S1 Work functions of TiN at different surface orientations, i.e. TiN(001), TiN(110) and TiN(111) with Ti/N as termination, respectively.

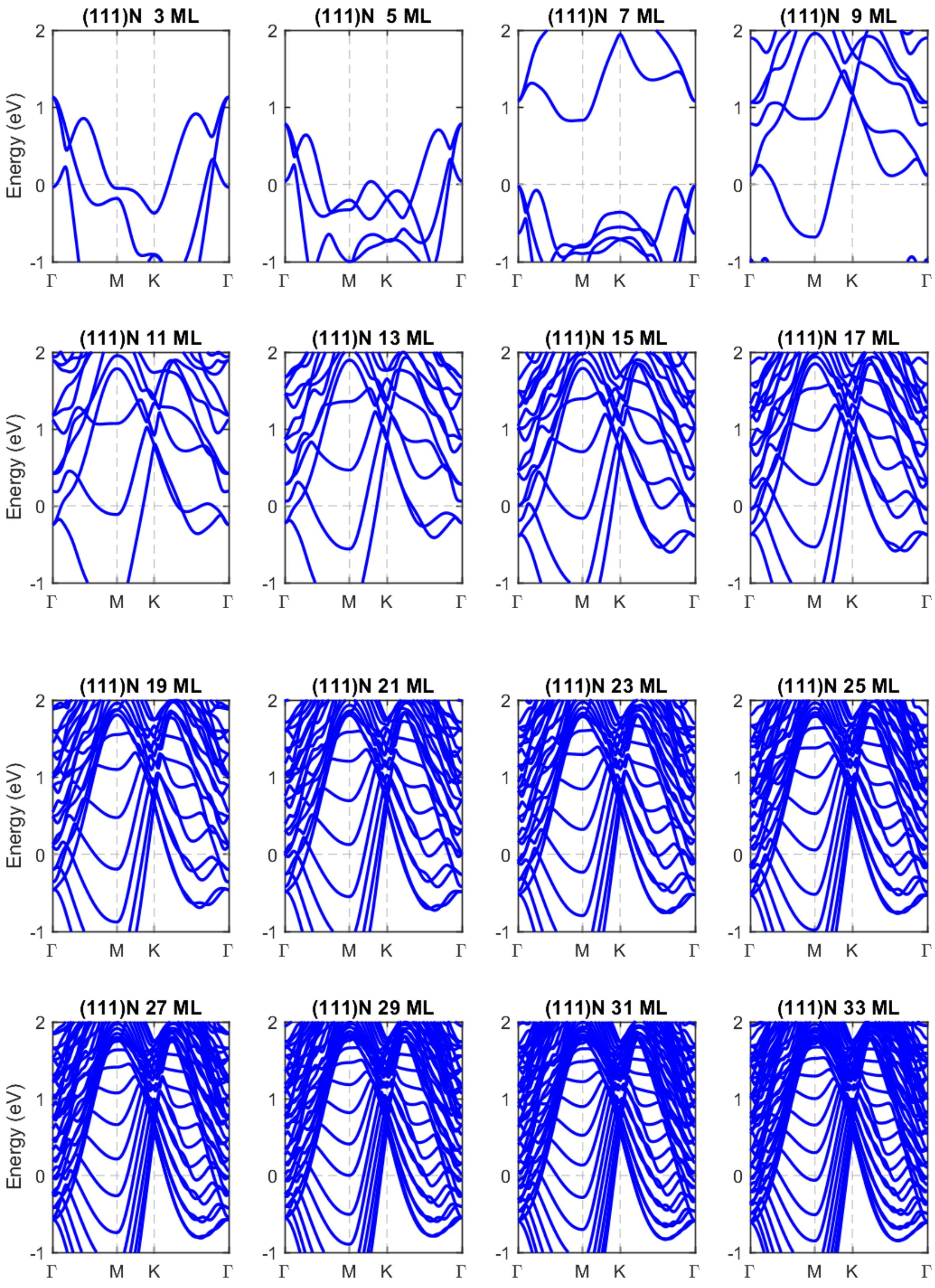

Fig. S1. Band structure of TiN (111) N-terminated thin film.

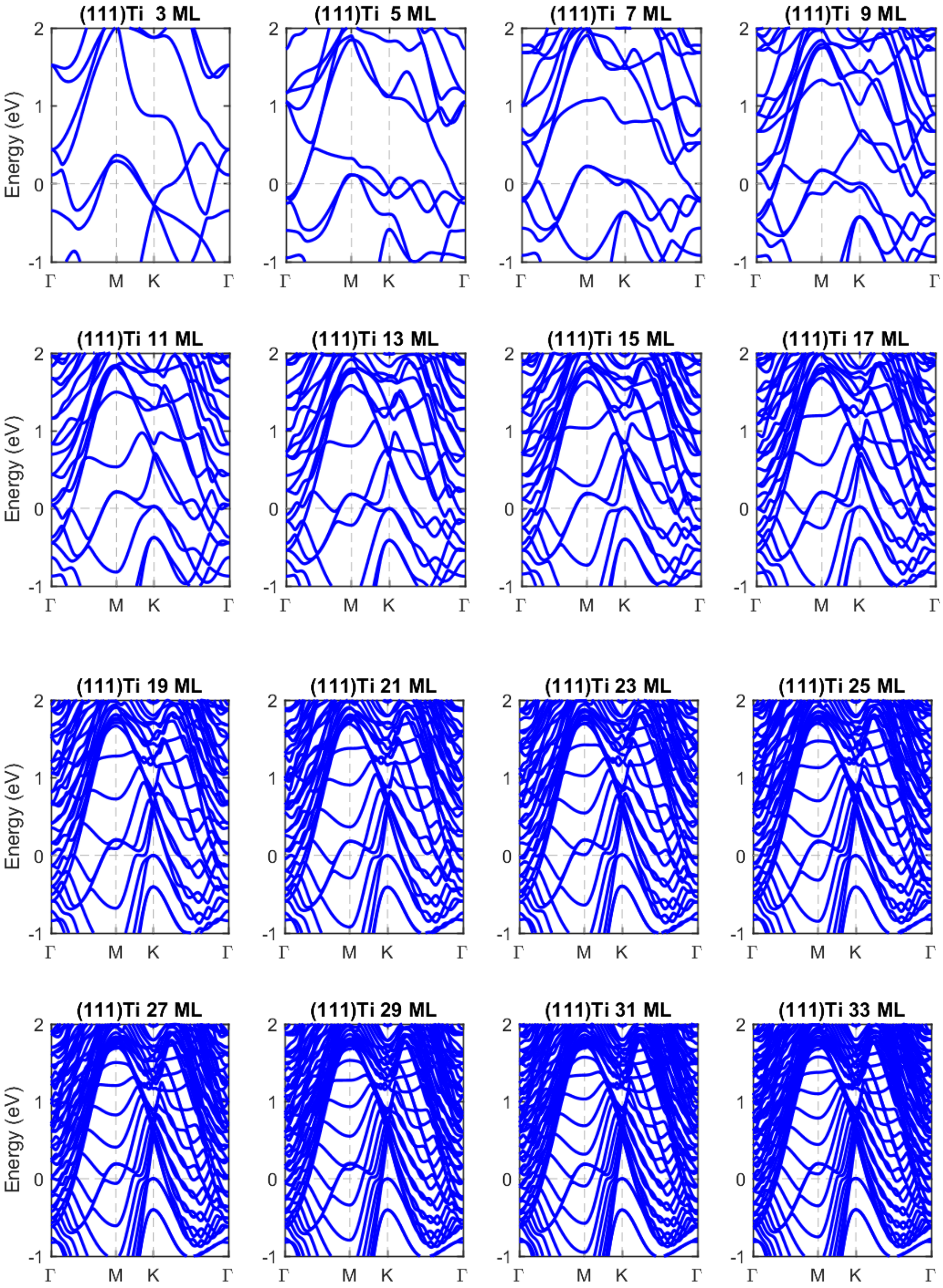

Fig. S2. Band structure of TiN (111) Ti-terminated thin film.

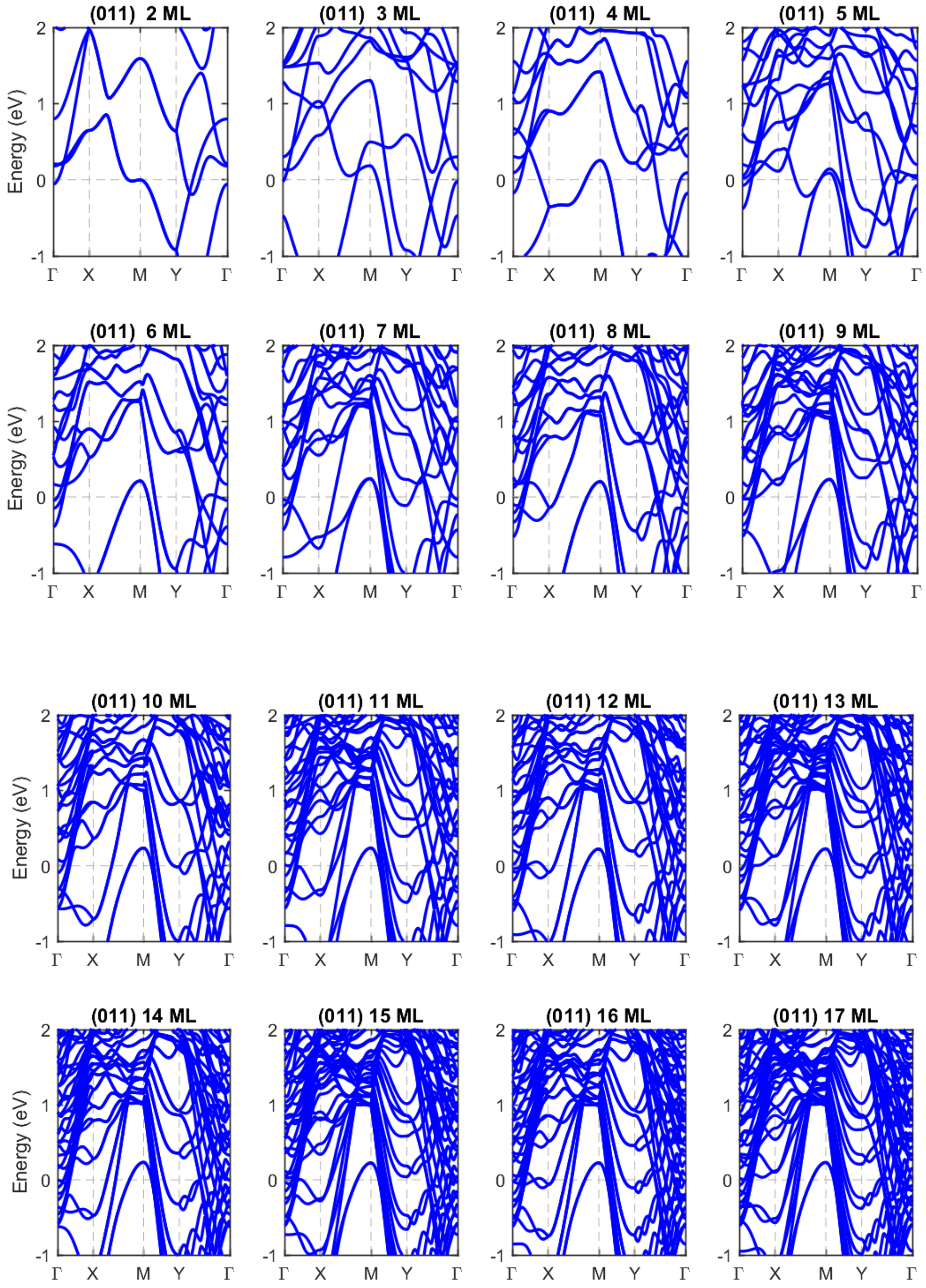

Fig. S3. Band structure of TiN (011) thin film.

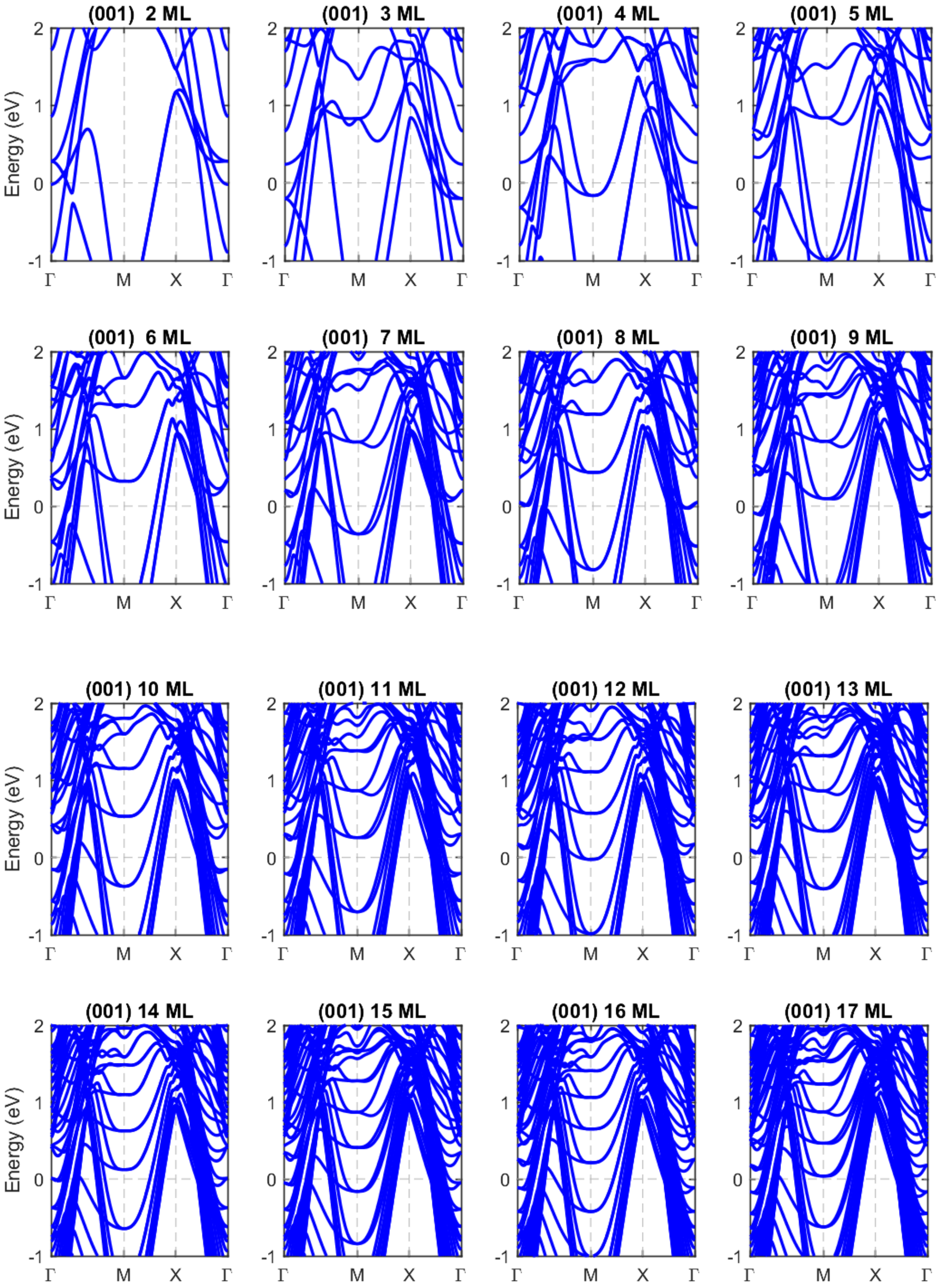

Fig. S4. Band structure of TiN (001) thin film.

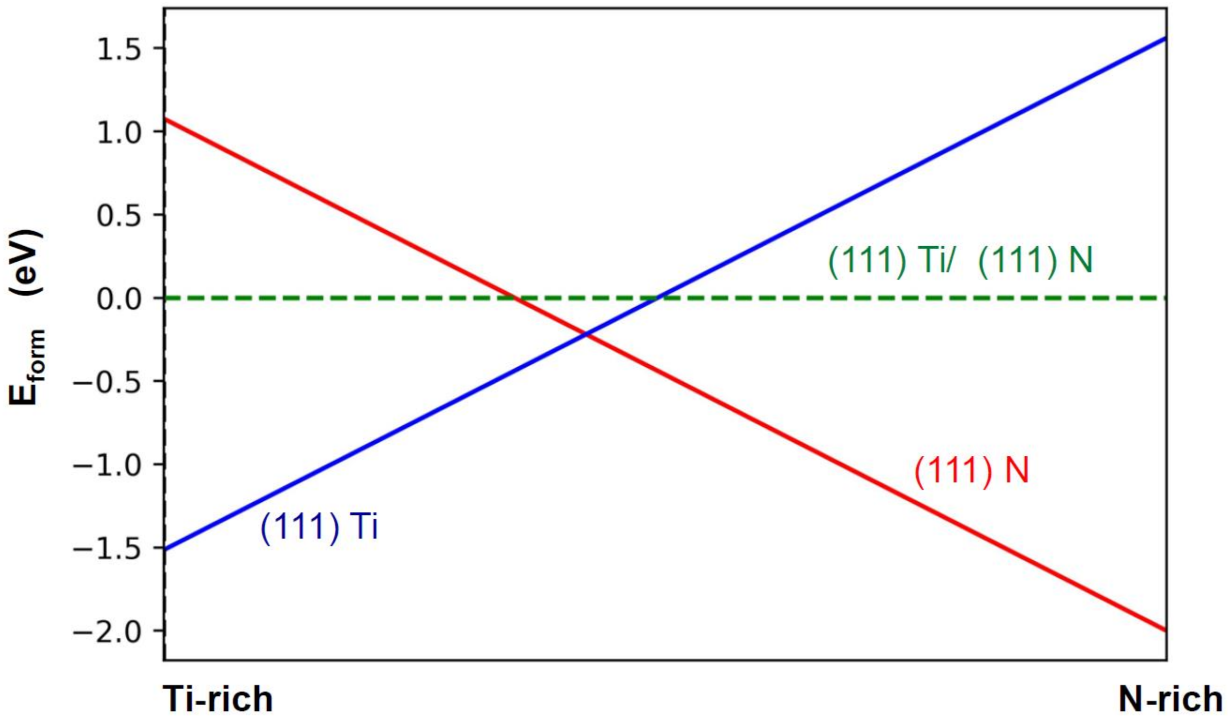

Fig. S5. Formation energy of surface with N-termination at both sides, (111) N; surface with Ti-termination at both sides, (111) Ti; surface with Ti/N-termination at opposite sides, (111)Ti/(111)N.

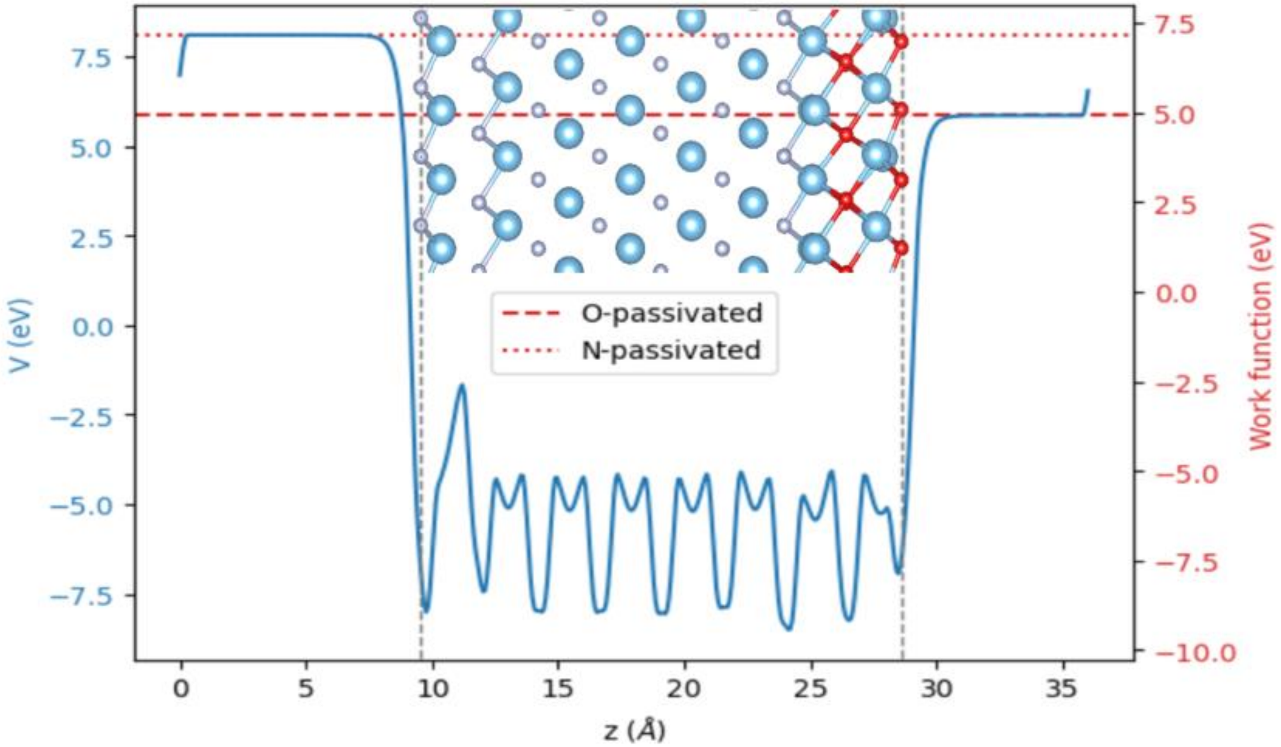

Fig. S6. Potential profile along the surface-normal direction (left axis) and work functions (right axis) of a (111) slab with N-passivated and O-passivated surfaces at opposite ends.

Surface Orientations	Work Functions (eV)
(001)	3.09
(110)	3.46
(111)Ti	4.74
(111)N	7.31

Table. S1 Work functions of TiN at different surface orientations, i.e. TiN(001), TiN(110) and TiN(111) with Ti/N as termination, respectively.
